# Structure and dynamics of the pan-genome of *Streptococcus pneumoniae *and closely related species

**DOI:** 10.1186/gb-2010-11-10-r107

**Published:** 2010-10-29

**Authors:** Claudio Donati, N Luisa Hiller, Hervé Tettelin, Alessandro Muzzi, Nicholas J Croucher, Samuel V Angiuoli, Marco Oggioni, Julie C Dunning Hotopp, Fen Z Hu, David R Riley, Antonello Covacci, Tim J Mitchell, Stephen D Bentley, Morgens Kilian, Garth D Ehrlich, Rino Rappuoli, E Richard Moxon, Vega Masignani

**Affiliations:** 1 Novartis Vaccines and Diagnostics, Via Fiorentina 1, 53100 Siena, Italy; 2 Allegheny General Hospital, Allegheny-Singer Research Institute, Center for Genomic Sciences, Pittsburgh, Pennsylvania 152123, USA; 3 Institute for Genome Sciences, Department of Microbiology and Immunology, University of Maryland School of Medicine, 801 West Baltimore Street, MD 21201, USA; 4 The Sanger Institute, Wellcome Trust Genome Campus, Hinxton, Cambridge CB10 1SA, UK; 5 Laboratorio di Microbiologia Molecolare e Biotecnologia, Dipartimento di Biologia Molecolare, Universita' di Siena, Policlinico Le Scotte, 53100 Siena, Italy; 6 Division of Infection and Immunity, Glasgow Biomedical Research Centre, University of Glasgow, 120 University Place, Glasgow G12 8TA, UK; 7 Institute of Medical Microbiology and Immunology, Aarhus University, DK-8000 Aarhus, Denmark; 8 University of Oxford Department of Paediatrics, Medical Sciences Division, John Radcliffe Hospital, Headington OX3 9DU, UK

## Abstract

**Background:**

*Streptococcus pneumoniae *is one of the most important causes of microbial diseases in humans. The genomes of 44 diverse strains of *S. pneumoniae *were analyzed and compared with strains of non-pathogenic streptococci of the Mitis group.

**Results:**

Despite evidence of extensive recombination, the *S. pneumoniae *phylogenetic tree revealed six major lineages. With the exception of serotype 1, the tree correlated poorly with capsular serotype, geographical site of isolation and disease outcome. The distribution of dispensable genes - genes present in more than one strain but not in all strains - was consistent with phylogeny, although horizontal gene transfer events attenuated this correlation in the case of ancient lineages. Homologous recombination, involving short stretches of DNA, was the dominant evolutionary process of the core genome of *S. pneumoniae*. Genetic exchange occurred both within and across the borders of the species, and *S. mitis *was the main reservoir of genetic diversity of *S. pneumoniae*. The pan-genome size of *S. pneumoniae *increased logarithmically with the number of strains and linearly with the number of polymorphic sites of the sampled genomes, suggesting that acquired genes accumulate proportionately to the age of clones. Most genes associated with pathogenicity were shared by all *S. pneumoniae *strains, but were also present in *S. mitis, S. oralis *and *S. infantis*, indicating that these genes are not sufficient to determine virulence.

**Conclusions:**

Genetic exchange with related species sharing the same ecological niche is the main mechanism of evolution of *S. pneumoniae*. The open pan-genome guarantees the species a quick and economical response to diverse environments.

## Background

*Streptococcus pneumoniae *is a major causative agent of human diseases, which include chronic otitis media, sinusitis, pneumonia, septicemia, and meningitis. While other pathogenic streptococci can be easily identified both phenotypically and through molecular phylogenetic analysis, *S. pneumoniae *is very similar to commensal species of the Mitis group, in particular *Streptococcus mitis, Streptococcus oralis *and *Streptococcus infantis *[1].

Most strains of these species can take up DNA from the environment and recombine sequences into their chromosome [2], resulting in both substitution of DNA fragments by homologous sequences from other clones and acquisition of novel genes from donor organisms, a process termed horizontal gene transfer (HGT). Due to the dynamic effects on genome content and organization resulting from HGT, it has been argued that the evolution of individual strains is substantially shaped by recombination-dependent novel acquisitions of DNA, commensurate with the genetic diversity of the species. The repertoire of genetic sequences of named species, such as *S. pneumoniae*, has been termed the pan-genome [3,4]. The maintenance of these HGT systems is particularly striking when viewed from a genomic perspective. Commensal and pathogenic bacteria that are exclusively adapted to a restricted range of hosts maintain relatively small genome sizes, in the range of 1.5 to 3 megabases, when compared to free-living environmental species. The reduction in genome size, reflecting evolutionary constraints on the retention and build up of nonessential genes, occurs despite the conservation of multiple operons that support HGT. It has been proposed that the ability of some bacterial commensal pathogens to generate diversity through HGT provides a selective advantage to these microbes in their adaptation to host econiches and evasion of immune responses [5,6].

Isolates of *S. pneumoniae *are traditionally characterized in terms of the chemical composition of their polysaccharide capsules, of which there are more than 90 serotypes. Different serotypes display different pathogenic potential and geographic distribution [7,8]. However, genomic variability among strains of *S. pneumoniae *is more accurately inferred from a comparison of allelic profiles of housekeeping genes [9], multi-locus sequence typing (MLST) [10], than by capsular serotyping [2,4]. This assertion has been strengthened by analyzing the distributions of the pilus-encoding *rlrA *and *PI-2 *islets in large collections of strains [11,12]. Although frequent recombination violates the paradigm of strict clonal inheritance, recently evolved clones maintain a high level of genomic similarity. This raises the question as to the extent of the distribution of dispensable genes, how these might be understood in terms of a clonal population structure and which genes, or classes of genes, violate this structure.

Most of these aspects of molecular evolution, traditionally addressed by population genetics methods, can be more advantageously studied by comparing whole genome sequences [3,13,14]. There are several complete genome sequences of individual strains of *S. pneumoniae *[15-20] and a comparative analysis of 17 complete and draft sequences predicted that the complement of genes that can be found in the genome of *S. pneumoniae *comprises more than 5,000 families of orthologs [4]. Here we report the genomic variability in 44 strains (24 newly sequenced and 20 already present in public databases) of *S. pneumoniae *in comparison with four newly sequenced genomes of *S. mitis *and one newly sequenced genome each of *S. oralis *and *S. infantis*. This genome scale study uses the most complete sampling of the diversity of the *S. pneumoniae *species to date, including the first analysis of multiple strains of the same serotype or MLST clonal complex (CC), to investigate the evolutionary processes that lead to the divergence of *S. pneumoniae *from other related commensal species.

Our analysis is divided in two parts. In the first section, we present data on the genomic variability of *S. pneumoniae *in the context of previous studies on the population structure of this species. In the second section we focus on genome dynamics, extending the analysis to the non-pathogenic streptococcal strains, and discuss possible evolutionary implications.

## Results and Discussion

### Genomic variability of *S. pneumoniae*

#### An average of 74% of any individual genome is shared by all strains

We aligned the genome sequences of 44 *S. pneumoniae *strains, 14 complete and 30 draft (Additional file 1). The collection spanned 19 different serotypes, 24 MLST CCs, as defined by the eBURST algorithm [21], and a set of laboratory (*n *= 1), disease-associated (*n *= 37) and carriage-associated (*n *= 6) strains isolated in different geographic locations. The sampled CCs accounted for 53% (1,715 0f 3,222) of the known sequence types (STs) as of March 2009 if singletons are excluded (that is, STs that are not part of any CC), or 42% (1,715 of 4,098) if singletons are included.

Excluding gap-containing aligned areas, the cumulative length of the alignment shared by all strains, the core genome, was 1,536,569 bp. Given an average genome length of 2,088,534 bp, on average 74% of each sequence is conserved by all strains. This core genome alignment had 79,171 polymorphic sites, of which 50,924 were informative, that is, the substitution was common to at least two sequences.

**Figure 1:**
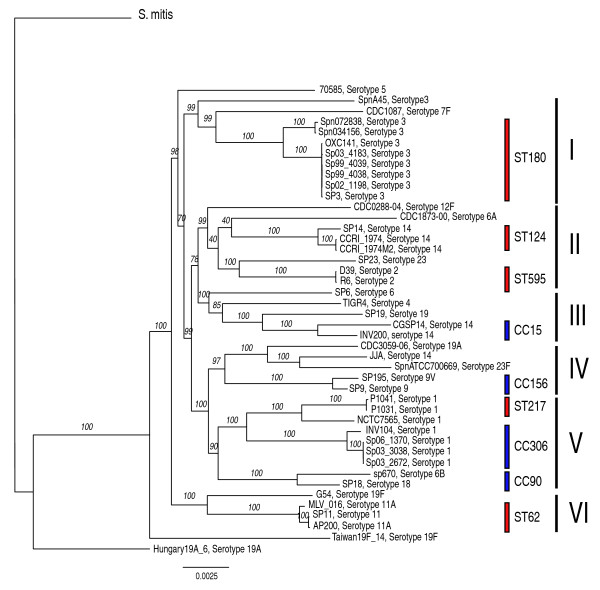
**Maximum likelihood phylogenetic tree obtained using the SNPs of the core genome of the 44 *S. pneumoniae *genomes**. The tree has been rooted using the four *S. mitis *genomes as outgroup, but note that the branch connecting the *S. pneumoniae *clade to the *S. mitis *clade is not to scale. The branches are annotated with their bootstrap support (numbers in italics). Red bars indicate strains belonging to the same sequence type (ST), while blue bars indicate strains belonging to the same clonal complex (CC). The six major lineages are identified by roman numbers I to VI.

Based on the polymorphic sites, we computed a maximum likelihood phylogenetic tree that was rooted using the *S. mitis *genomes as outgroup (Figure 1). This phylogeny had high bootstrap values in both the inner and outer branches. Strains of the same ST or CC were monophyletic. In addition, six major monophyletic lineages (I to VI in Figure 1) that included closely related STs or CCs were identified. While details on the mutual relationships between the lineages varied depending on the tree reconstruction method tested, the lineages themselves were supported with high confidence from alternative analyses (Additional files 2 and 3). Since CCs always formed monophyletic branches in the genome-based tree and given the coverage of our strain collection and of the MLST database, we estimate that about half of the circulating strains of *S. pneumoniae *fall into one of the six lineages.

To quantify the correlation between phylogeny and genome composition, we measured the average association value V [22] (see Materials and methods) of lineages I to VI and the CCs with the presence/absence of dispensable genes (that is, genes present in more than one strain but not in all strains; Additional file 4). Values of V = 0.5 for lineages and V = 0.82 for CCs were obtained. An even stronger association was found between the allelic form of core genes and the classification into lineages I to VI and CCs (V = 0.747 and V = 0.94, respectively).

In general, the position on the tree (Figure 1) did not reveal patterns predictive of whether strains were associated with carriage or disease, nor their geographical site of isolation. In particular, the eight strains isolated at a single institution in a short time window [4] were distributed randomly across the tree, supporting a model of global circulation of pneumococcal strains.

#### Frequent recombination distorts but does not obliterate phylogenetic signals of descent from a common ancestor

**Figure 2:**
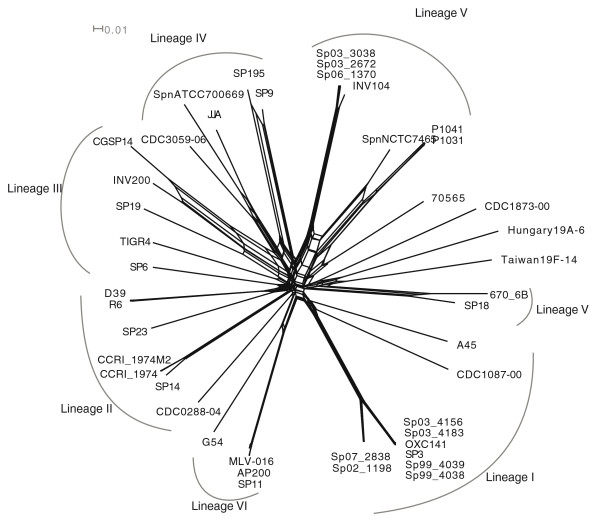
**Split network obtained using the SNPs of the core genome to depict the impact of recombination on 44 *S. pneumoniae *strains**. In this representation, all the conflicting phylogenetic signals due to each SNP are represented as alternative bipartitions that account for the non-tree-like structure of the inner part of the network. The six lineages highlighted in Figure 1 are also indicated.

Most *S. pneumoniae *strains and other related species are naturally competent, that is, they can take up genetic material from the environment and recombine it into their chromosome [2,13,23], weakening the phylogenetic signal contained in sequence alignments. To determine the effect of homologous recombination on the phylogeny, we used split networks [24] to visualize the contrasting phylogenetic signals (Figure 2). The main groups highlighted in Figure 1 are confirmed by the network analysis. Lineage V is split into two subgroups, one composed of the serotype 1 strains, and the other composed of the two serotype 6B CC90 strains (670-6B and SP18), which appear to be more similar to Taiwan19F-14. The role of recombination was evident from the non tree-like structure of the inner connections between the different lineages, the presumed consequence of DNA exchange amongst unrelated strains. However, the long branches separating groups of strains closely related to the lineages (I to VI) of Figure 1 support the idea that, while the inner structure of the inferred genealogy of *S. pneumoniae *is heavily influenced by recombination, molecular phylogenetic methods based on whole genomes are able to correctly reconstruct recent genealogical relationships.

#### Frequent recombination disrupts associations between capsular serotypes and clonal complexes, except for serotype 1

Poor correlation was observed between the serotype of a strain and its position in the tree. The notable exceptions were strains of serotypes 1 and 3, which formed two monophyletic branches. However, while all serotype 3 strains (except SpnA45) were of a single ST (ST180), serotype 1 strains constituted three major lineages belonging to a single cluster with significant bootstrap support. These lineages represent the three CCs (CC217, CC306 and CC2296) of circulating serotype 1 strains that are associated with distinct geographical areas [25]. The robustness of our sampling of serotype 1 was supported by an analysis of the MLST database (see Materials and methods). The three CCs present in our dataset accounted for 87% of all serotype 1 strains, and five CCs made up 97%, a situation very different from other serotypes that were much more heterogeneous in terms of genotype composition. For comparison, 97% coverage required 12 CCs for serotype 3, 14 CCs for serotype 14, and 27 CCs for serotype 19F.

We tested the hypothesis that genetic exchange in serotype 1 strains is restricted by estimating the fraction of 680 core genes that displayed evidence of recombination (see Materials and methods) in the serotype 1 strains. We found evidence of recombination in 205 of 680 loci, suggesting that the correlation between capsular serotype and position on the tree cannot be attributed only to the low probability of exchange of genetic material with strains of different serotypes.

#### The pan-genome of *S. pneumoniae*

Sequence variability can be described from static and dynamic points of view. While a description of the dynamics requires a realistic model of the relevant evolutionary processes, here we report a static description that provides a synthetic representation of the genome variability of the species in terms of a few parameters. We calculated the size of the total *S. pneumoniae *gene pool accessible to the species, or pan-genome, using two different methodologies, namely the finite supragenome model [26] and the power law regression model [3,27].

**Figure 3:**
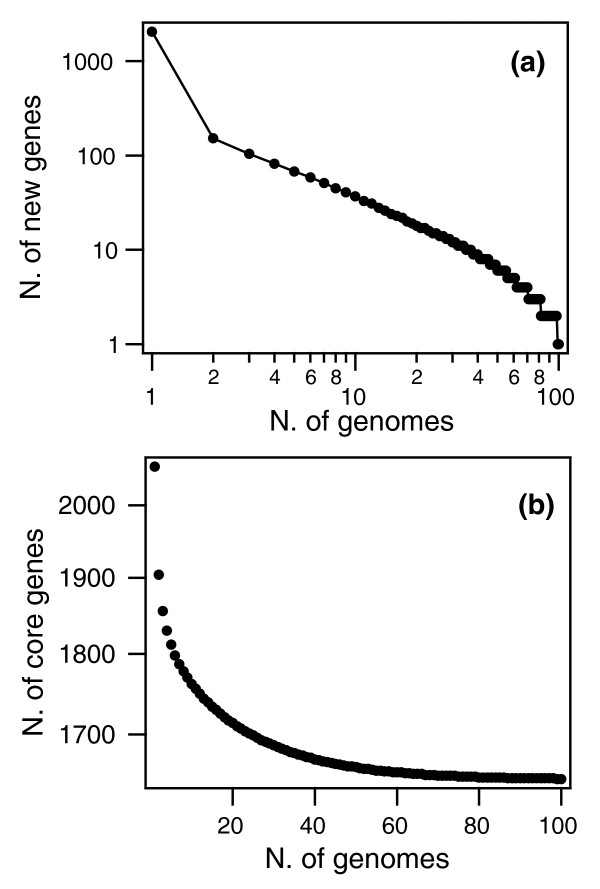
**The *S. pneumoniae *pan-genome according to the finite supragenome model**. **(a) **Number of new genes as a function of the number of sequenced genomes. The predicted number of new genes drops sharply to zero when the number of genomes exceeds 50. **(b) **Number of core genes as a function of the number of sequenced genomes. The number of core genes converges to 1,647 for number of genomes *n*→∞.

The finite supragenome model allows prediction of the number of genes present in a given fraction of the circulating strains, varying from rare genes (less then 3% of the strains) to core genes (all the strains). Based on the 44 sequenced strains, giving a total of 3,221 clusters (Table 1), the number of core, dispensable and total genes that would be expected for a 100-strain comparison was estimated. The model predicted a strong decline in the number of new genes identified (1 per genome at 100 strains) (Figure 3a) and stabilization in the number of core genes at 1,647 (Figure 3b). The supragenome model predicted that 48% of the genes are core and approximately 27% are rare, that is, present in less than 3% of the strains. Given that, by construction, this model predicts a finite number of genes in the species, the maximum likelihood size of the pan-genome was estimated to be 3,473 genes (range 3,300 to 5,000; see Materials and methods). Thus, we estimate that the 44 strains taken together encompass 92.7% (3,221 of 3,473) of the pneumococcal pan-genome.

**Table 1:** Number of clusters of orthologous genes

	*S. pneumoniae*	*S. pneumoniae , S. mitis, S. oralis, S. infantis*	*S. pneumoniae , S. mitis, S. oralis, S. infantis, S. Sanguinis, S. pyogenes*
Number of genes	3,221	4,904	7,031
Core	1,666 (52%)	1,111 (23%)	522 (7%)
Dispensable	1,555 (48%)	3,793 (77%)	6,509 (93%)
Strain-specific	389 (12%)	1,565 (32%)	3,552 (51%)

We report the total number of clusters of orthologous genes and the number of core, dispensable (that is, missing in at least one strain), and strain-specific genes for *S. pneumoniae *alone, for *S. pneumoniae, S. mitis, S. oralis*, and *S. infantis*, and for *S. pneumoniae, S. mitis, S. oralis, S. infantis, S. sanguinis *and *S. pyogenes.*

**Figure 4:**
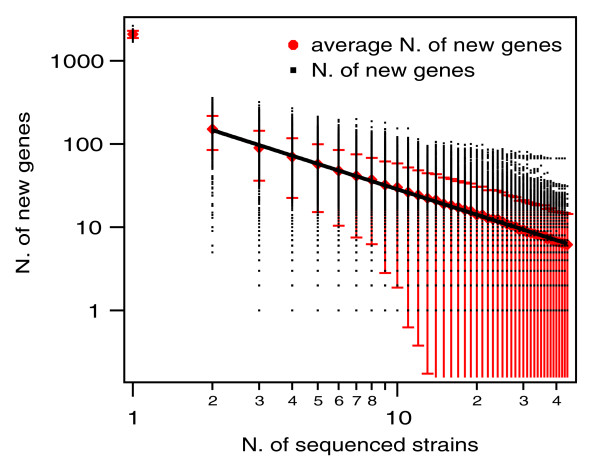
**The *S. pneumoniae *pan-genome according to the power law model**. The number of specific genes is plotted as a function of the number (n) of strains sequentially added (see Materials and methods). For each n, points are the values obtained for the different strain combinations; red symbols are the average of these values, and error bars represent standard deviations. The superimposed line is a fit with a decaying power law *y *= *A*/*n*^*B*^. The fit parameters are *A *= 295 ± 117 and *B *= 1.0 ± 0.15.

In contrast to the supragenome model, the power law regression model [3] (Figure 4) allowed the extrapolation to an infinite number of strains, providing a prediction of whether the number of distinct genes that can be found in *S. pneumoniae *is finite (closed pan-genome) or unlimited (open pan-genome). A comparison of the data from Figures 3 and 4 showed that, for an intermediate number of genomes (<40) the predictions of the two models were consistent. For large numbers of genomes (>40), the finite supragenome model sharply goes to zero, while in the regression model the average number of new genes as a function of the number of genomes is well described by a power law, with a fitted exponent *ξ *= -1.0 ± 0.15 (Figure 4). Thus, the pan-genome is open, its size increasing logarithmically, findings that position the pneumococcal species on the edge between an open (*ξ >*-1) and closed (*ξ <*-1) pan-genome (see Materials and methods).

**Figure 5:**
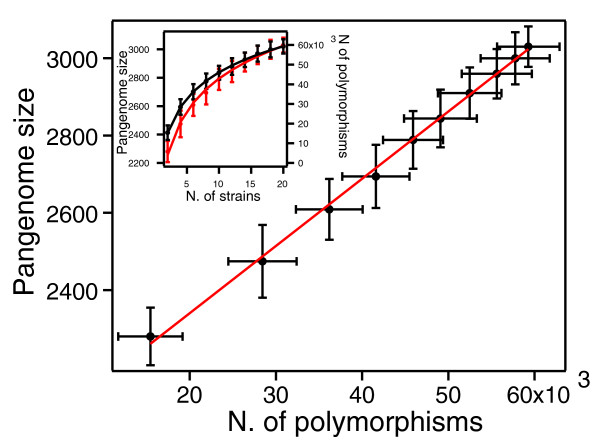
**Size of the pan-genome versus the number of polymorphic sites**. The slope of the fitted line gives the ratio between the rate of acquisition of new genes and the population mutation rate *ω*/*θ *= 0.017 ± 0.0017. In the inset, the size of the pan-genome (red dots) and number of polymorphic sites (black dots) as a function of the number of genomes are shown. The lines are least squares fit with a logarithmic law. The error bars represent the standard deviation of the data.

To investigate how the size of the pan-genome is related to the genetic diversity within the sample of strains, and to estimate how the rate of acquisition of new genes compares to the mutation rate, the pan-genome size was plotted versus the number of polymorphic sites for samples of different sizes (Figure 5; see Materials and methods). The results show a linear correlation between these two quantities. This result can be explained if we assume that new genes and mutations are acquired by pneumococci with constant rates (*ω *and *θ*, respectively) over time. While these parameters cannot be estimated separately from the data, from the slope of a linear fit of the two quantities plotted one versus the other (Figure 5), the ratio *ω/θ *between the rate of acquisition of new genes and the population mutation rate can be estimated: *ω/θ *= 0.017 ± 0.002. This result indicates that, on average, a new gene is acquired by the population every 59 mutations.

### Genome dynamics and evolution

#### Dispensable sequences are recent acquisition events, and are frequently transferred among strains

**Figure 6:**
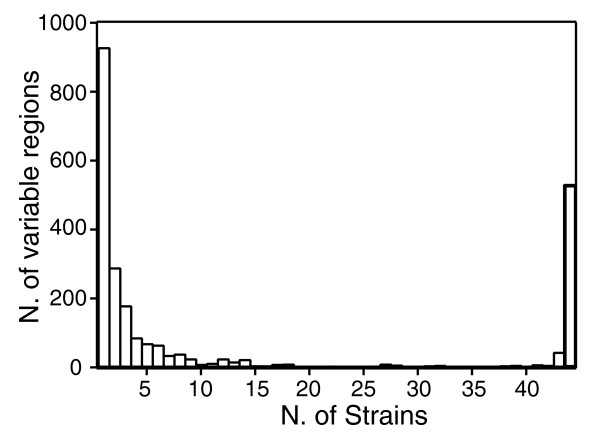
**Histogram of the number of genomes sharing variable regions of size greater than 500 bp**. The distribution is bimodal, with most of the variable regions either being present in most of the strains, or being present only in a small number of strains.

To investigate how the dispensable genome is distributed in the pneumococcal population, the frequency distribution of the genome segments that were absent from at least one strain was studied. To avoid bias and give equal weight to all acquisition and loss events, we selected the 1,030 genomic regions longer than 500 bp that were not present in all strains, irrespective of the number of genes that they encoded (see Materials and methods). Figure 6 depicts a histogram counting the number of strains sharing a particular region. The distribution is bi-modal, since the variable regions were present either in most of the 44 strains, likely representing a recent deletion, or in a small proportion of strains (less than 10), probably including recent acquisition events.

**Figure 7:**
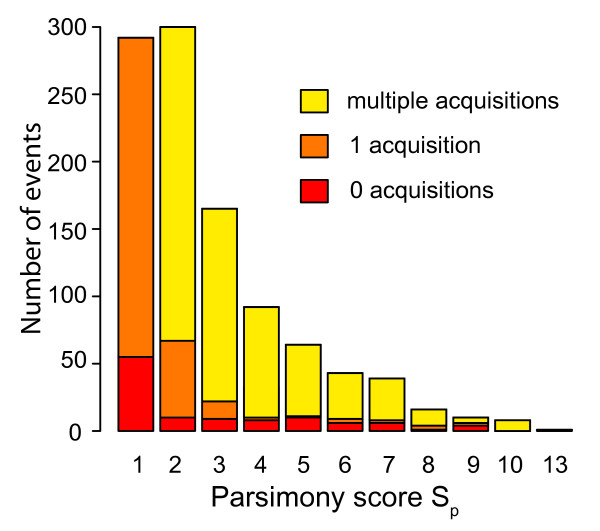
**Histogram of the parsimony score *S*_*p *_of the presence/absence of the variable regions of size greater than 500 bp, computed for the tree shown in Figure 1**. For a given dispensable region, *S*_*p *_represents the number of acquisition and loss events (*S*_*p *_= *N*_*a *_+ *N*_*l*_, where *N*_*a *_and *N*_*l *_are the number of acquisitions and losses, respectively) required for its pattern of presence/absence on the tree in Figure 1. The colors indicate the number of acquisitions *N*_*a*_, while the number of losses can be calculated as *N*_*l *_= *S*_*p *_-*N*_*a*_. For simplicity, all segments with *N*_*a *_> 1 have been collapsed in a single bar. Since an acquisition followed by a recombination event can always be explained by multiple acquisitions, events with *N*_*a *_> 1 are possible intra-species recombination events.

To gain insight into the dynamics of the dispensable genome, the most parsimonious pattern of acquisitions and losses compatible with the tree in Figure 1 was reconstructed for each variable region. In Figure 7 we show a histogram counting the number of segments that have undergone a given number of acquisition or loss events during the evolution of the species. The columns are partitioned by the number of acquisitions. Of the 1,030 selected regions, 109 segments were present in the ancestral genome and were subsequently lost by some strains (red bars in Figure 7); 321 segments were acquired once (orange bars in Figure 7); while the remaining 600 segments have undergone repeated acquisitions, suggesting that they encode highly mobile elements (yellow bars in Figure 7).

On the basis of these data, the presence or absence of dispensable regions can be used to discriminate only recently diverging groups of strains, whereas they give a much weaker signal for older differentiation events, indicating that the dispensable genome composition cannot resolve the inner structure of the phylogenetic tree of the species (Additional file 5).

#### Linkage disequilibrium patterns demonstrate that recombination proceeds through gene conversion

**Figure 8:**
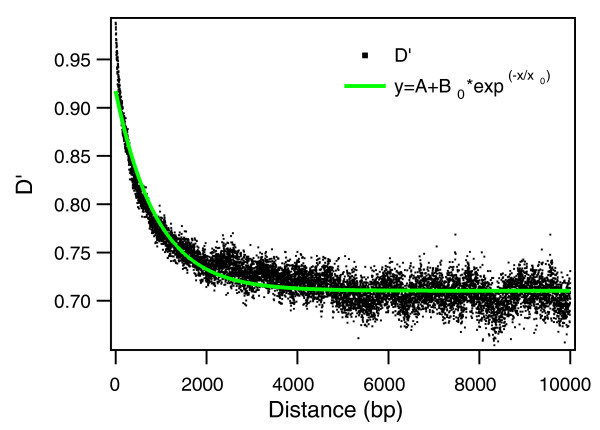
**Average value of D' plotted as a function of the distance (in base pairs) along the chromosome between the pairs of polymorphic sites**. The green line is a least-square fit with the exponential function *y *= *A *+ *Be*^*-x/x0*^, with *A *= 0.07103 ± 0.0002, *B *= 0.201 ± 0.001 and *x*_0 _= 896 ± 7.

Despite the presence of widespread recombination, the persistence of a detectable phylogenetic signal in whole genome alignments indicates that recombination, although frequent, did not completely obscure the non-random association of polymorphisms at distant loci. To further investigate this phenomenon, the correlation between polymorphisms at different loci, or linkage disequilibrium (LD), was characterized by measuring the Lewontin's D' parameter [28] as a function of the distance along the chromosome (Figure 8). D' quickly converged to a plateau, as expected under a gene conversion model, where exchange of DNA sequences occurs through the substitution of short stretches of DNA with homologous DNA from a different cell. An exponential fit to the data (green line in Figure 8) revealed a decay length *x*_0 _= 896 ± 7 bp, and a plateau value *A *= 0.7103 ± 0.002. These results do not change appreciably if closely related strains are excluded from the analysis by retaining only one representative strain for each ST.

The value of the characteristic length of the recombining segments, close to the average length of genes (855 bp in the sequenced strains), is probably the result of a bias towards events in which entire genes are exchanged, and is similar to the estimates previously obtained in a different species [29]. The relatively high value of the D' plateau indicates that recombination, although frequent, did not completely obscure the non-random association of distant alleles, implying that long sequences contain a coherent phylogenetic signal even in the presence of recombination, as recently found by simulations [14].

To quantify the relative contribution of mutation and recombination to sequence variability, for each of the locally collinear blocks (LCBs) of the core alignment we have computed the per-site Watterson mutation rate *θ *and per-site recombination rate *ρ*, which is the per-site probability of a recombination breakpoint, using LDhat [30]. There was a considerable spread in the values of both *θ *and *ρ *for the different regions of the alignment. *θ *ranged between 7.06 × 10^-5 ^and 0.019, with an average of 0.0032, while *ρ *ranged between 9.3 × 10^-5 ^and 0.98, with an average of 0.018. The average value of the ratio *ρ*/*θ *between the recombination rate *ρ *and the mutation rate *θ *was equal to 5.57. We estimated the relative impact of recombination and mutation in generating the sequence diversity of *S. pneumoniae *by computing the rate *θ*_*ρ *_at which a mutation is introduced by a recombination event and the ratio *θ*_*ρ*_/*θ*, where *θ *is the mutation rate. *θ*_*ρ *_was estimated using the formula *θ*_*ρ *_= *θ·ρ·l*, where *l *is the average length of the recombining segments, and *θ *and *ρ *are the mutation and recombination rates, respectively. Considering *l = 896 *bp, as determined from the linkage disequilibrium decay length (Figure 8), we estimated that the rate *θ*_*ρ *_was *θ*_*ρ *_= *θ·ρ·l = *0.052, and the average value of the ratio *θ*_*ρ*_/*θ *was *θ*_*ρ*_/*θ *= 16.2, to be compared with the previous estimate of *θ*_*ρ*_/*θ = *50 obtained for housekeeping genes [31].

#### *S. pneumoniae *is closely related to *S. mitis*

To gain insight into the differences underlying closely related pathogenic and non-pathogenic streptococcal species, the comparative analysis performed on the 44 genomes of *S. pneumoniae *was extended to include four *S. mitis*, one *S. oralis *and one *S. infantis *strains (Additional file 1).

**Figure 9:**
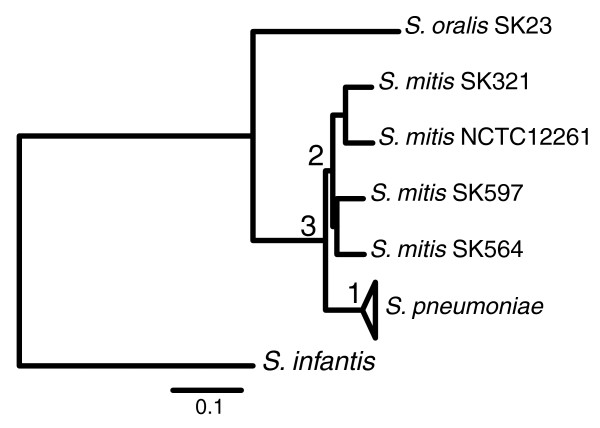
**Maximum likelihood phylogenetic tree obtained using the SNPs of the core genome of the 44 strains of *S. pneumoniae*, 4 strains of *S. mitis *and 1 strain each of *S. oralis *and *S. infantis***. For clarity the clade containing the *S. pneumoniae *strains has been collapsed. The numbers on the internal nodes label the last common ancestor of the *S. pneumoniae *species (1), of the *S. mitis *species (2), and of the *S. pneumoniae*-*S. mitis *complex (3).

The whole genome alignment of the 50 strains was computed. Excluding gaps, 998,057 bp of each sequence can be aligned against all other sequences, representing, on average, 48% of the pneumococcal genomes, 51% of the *S. mitis *genomes (average genome length of 1,949,224 bp), and 53% and 55% of the *S. oralis *and *S. infantis *genomes, respectively. Of these, 283,596 positions were polymorphic. A phylogenetic tree based on these polymorphic sites (Figure 9, where the *S. pneumoniae *branch is collapsed for readability) revealed that the most divergent species from *S. pneumoniae *is *S. infantis*, followed by *S. oralis*, while the *S. mitis *strains are the most closely related to *S. pneumoniae*. Interestingly, while the average genetic distance among the *S. pneumoniae *strains was 0.010 ± 0.001, the same value calculated among *S. mitis *strains was much larger (0.066 ± 0.007), and only slightly smaller than that between strains of *S. mitis *and *S. pneumoniae *(0.081 ± 0.010), confirming the high variability of the *S. mitis *species [1] and suggesting that *S. pneumoniae *is a pathogenic and epidemiologically successful clone of a larger distinct and coherent population that includes the numerous *S. mitis *lineages.

#### The core and pan-genome analysis of the *S. pneumoniae*-*S. mitis *complex supports the close relationship between the two species

**Figure 10:**
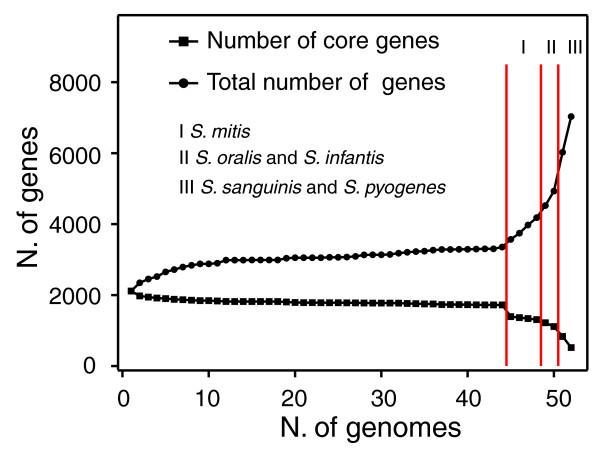
**Variation of the number of dispensable and core genes upon the addition of new species or strains**. Strains are added sequentially, starting with the 44 *S. pneumoniae *strains followed by the *S. mitis *(region I), *S. oralis *and *S. infantis *(region II), *S sanguinis *and *S. pyogenes *(region III) strains.

To characterize the differences in the genomic composition between the different species, we computed the set of cluster of ortholog genes shared by all *S. pneumoniae *isolates and by *S. pneumoniae, S. mitis, S. infantis*, and *S. oralis *(Table 1). In total, these four species contained 4,904 clusters of orthologs, of which 1,111 are present in all strains. We investigated how the size of the pan- and core genome is influenced by the addition of strains belonging to closely related species (Figure 10). The addition of the first *S. mitis *strain contributed approximately 200 new genes to the pneumococcal pan-genome. As an additional three strains were added, each introduced approximately 200 new genes, providing further evidence for the high variability of the dispensable part of the genome of *S. mitis*.

On the other hand, as might be expected for the addition of a different species, the first *S. mitis *strain caused a drop in the core genome from approximately 51% to approximately 39% of the total clusters, showing that some of the essential pneumococcal genes are not core in *S. mitis*. Yet, additional *S. mitis *strains had a minimal effect on the core genome, reflecting stabilization in the number of core genes shared between these species. The effect of the *S. mitis *strains on the streptococcus pan-genome calculation stood in stark contrast to the effect of *S. pyogenes *and/or *S. sanguinis *strains, since addition of any of these added over 1,000 new genes and caused a sharp drop in the core genome to less then 14% of the total genes. The difference in magnitude between the increase in variability observed from the inclusion of *S. mitis *strains relative to that observed with *S. sanguinis *or *S. pyogenes *strains highlights the close relationship between *S. pneumoniae *and *S. mitis*.

#### *S. mitis *evolved by genome reduction from its common ancestor with *S. pneumoniae*

To gain insight into the speciation of the *S. pneumoniae*-*S. mitis *complex, we inferred the genomic content of the common ancestors of *S. pneumoniae *(node 1 in Figure 9), *S. mitis *(node 2 in Figure 9) and of the *S. pneumoniae*-*S. mitis *complex (node 3 in Figure 9) using maximum likelihood. According to this reconstruction, the ancestral genome of all *S. pneumoniae *strains was composed of 2,281 genes, while the ancestral genome of *S. mitis *was composed of 1,888 genes, and the genome of the common ancestor of *S. pneumoniae *and *S. mitis *encoded 2,039 genes. For comparison, the average number of genes of *S. pneumoniae *is 2,104, and the average number of genes in *S. mitis *is 1,900. Although probably biased by the different number of sequenced *S. mitis *and *S. pneumoniae *strains, these results indicate that while the size of the *S. mitis *genomes has reduced since their diversification from *S. pneumoniae*, the ancestral *S. pneumoniae *genome has grown since its diversification from *S. mitis *while contemporary *S. pneumoniae *strains are now in a process of genome reduction. Interestingly, *S. pneumoniae *strains were more closely related to the ancestor of *S. mitis *(node 2 in Figure 10) than to the contemporary sequenced *S. mitis *strains themselves. Indeed, while contemporary *S. pneumoniae *strains shared between 71% (Hungary19A_16) and 67% (CDC0288) of their genome with the reconstructed ancestral *S. mitis *genome (node 2 in Figure 9), contemporary *S. mitis *strains conserved only between 67% and 64% of the genome of their common ancestor. These observations support the recently proposed theory that *S. mitis *evolved by genome reduction from a bacterium closely related to *S. pneumoniae *[1].

#### *S. mitis *and other streptococci are the main reservoir of genetic variability for *S. pnemoniae*

*S. pneumoniae *and *S. mitis *are known to colonize the same ecological niche and to actively exchange genetic material [32]. It is therefore interesting to quantify the degree by which homologous recombination and HGT between these two species contributed to the evolution of their core and dispensable genomes.

To estimate the fraction of the genome shared by *S. pneumoniae *and *S. mitis *that has been exchanged by homologous recombination, we have computed the number of bi-allelic SNPs in the multiple alignment of the 50 genomes that are polymorphic both in *S. pneumoniae *and in *S. mitis*. We found that of the 49,670 SNPs in *S. pneumoniae *(107,602 in *S. mitis)*, 14,655 were bi-allelic also in *S. mitis*. Although the directionality of the exchange events cannot be established, these data suggest that as much as 30% of the sequence variability in the part of the genome of *S. pneumoniae *shared with *S. mitis *could be due to homologous recombination with the latter. This fraction is likely to be an underestimate, due to the small number of sequenced *S. mitis *strains.

In order to identify the most likely origin of genes recently acquired by *S. pneumoniae *and to estimate the contribution of HGT between *S. pneumoniae *and *S. mitis *to the evolution of the dispensable genome of the former, all dispensable genes present in less than 50% of the *S. pneumoniae *strains were searched against a database of 792 complete bacterial genomes, supplemented by the newly sequenced *S. mitis, S. oralis *and *S. infantis*. The choice to restrict the analysis to genes present in a minority of the sequenced strains is aimed at minimizing the probability that these genes were present in the ancestor of *S. pneumoniae *and lost by some lineages, although this possibility cannot be ruled out. To select only recent acquisitions, hits with >90% identity over >90% of the sequence of the query gene were considered. Of 1,286 genes, only 16% (200) had a hit satisfying the cutoff. The vast majority of these (183 of 200) shared the highest homology with other streptococci. In particular, 62% (113) of the hits are in at least one of the *S. mitis *strains, followed by 31 hits in *Streptococcus suis*, 24 in *Streptococcus pyogenes*, 5 in *Streptococcus agalactiae *and *S. oralis*, 4 in *S. infantis *and 1 in *Streptococcus gordonii*. Hits outside the *Streptococcus *genus are: 7 in *Finegoldia magna*, 3 in *Staphylococcus aureus*, 2 in *Staphylococcus epidermidis*, 2 in *Macrococcus caseolyticus*, and 1 in each of *Clostridium difficile, Enterococcus fecalis*, and *Lacobacillus reuteri. *These data demonstrate that although most genes acquired by *S. pneumoniae *come from an unknown source, most of the genes with hits to the 792 complete bacterial genomes appear in other streptococci, and in particular in *S. mitis*, although HGT between distant species is also occasionally possible. Given the high variability of the *S. mitis *species, it is possible that many of the remaining 1,086 genes of unknown origin were acquired from *S. mitis *strains not yet sequenced, or more generally, from other still unknown bacterial species.

#### Strain distribution of genes involved in host-pathogen interaction and virulence

To elucidate the relationship between virulence potential and the evolution of *S. pneumoniae*, we have determined the distribution and conservation level of a set of 47 proteins that are either surface exposed, or known to be involved in interaction with the host and virulence [15]. The panel of selected proteins includes the entire set of LPXTG cell wall anchored molecules, and the choline binding proteins, a family of surface proteins that are specific to pneumococci and that are involved in bacterium-host cell adhesion [33]. Furthermore, we have added pneumolysin (Ply), a cholesterol-dependent cytotoxin implicated in multiple steps of pneumococcal pathogenesis [34,35], and a number of proteins that have been investigated as potential vaccine candidates [36], including the histidine triad proteins PhtA, B, D, E [37], PpmA [38], PsaA [39], PppA [40], and PcsB and StkP [41]. The distribution and level of conservation of each of these proteins within pneumococcal strains and the presence in *S. mitis, S. oralis *and *S. infantis *are reported in Table 2 and Additional file 6.

**Table 2:** Presence, cellular localization and level of conservation of a selection of genes that are either surface exposed, or known to be involved in interaction with the host and virulence

TIGR ID	R6 Id	INV104B	Length	Annotation	Predicted cell localization	Presence in Spn strains	Conservation (average % ID)	**Present in *S. mitis***^**a**^	Present in *S. oralis*	Present in *S. infantis*
Frameshifted^b^	spr1536		1,035	Neuraminidase A (NanA)	LPXTG cell-wall anchored	44	92.59	Yes	Yes	Yes
SP_0057	spr0057		1,312	Beta-N-acetylhexosaminidase (StrH)	LPXTG cell-wall anchored	44	97.59	No	Yes	Yes
SP_0069	Absent		211	Choline binding protein I (CbpI)	Choline binding, surface	6	85.73	Yes	No	No
SP_0071	Absent		1,856	Zinc metalloprotease ZmpC	LPXTG cell-wall anchored	6	89.79	Yes	No	No
SP_0082	spr0075		857	Cell wall surface anchor family protein	LPXTG cell-wall anchored	44	91.92	Yes	Yes	Yes
SP_0117	spr0097		744	Pneumococcal surface protein A (PspA)	Choline binding, surface	44	81.35	No	No	No
SP_0268	spr0247		1,280	Alkaline amylopullulanase, putative	LPXTG cell-wall anchored	44	97.53	Yes	Yes	No
SP_0314	spr0286		1,066	Hyaluronidase (HysA)	LPXTG cell-wall anchored	44	99.38	No	No	No
SP_0368	spr0328		1,767	Cell wall surface anchor family protein	LPXTG cell-wall anchored	44	96.96	No	Yes	Yes
SP_0377	spr0337		340	Choline binding protein C (CbpC)	Choline binding, surface	31	93.86	Yes	Yes	No
SP_0378	Absent		332	Choline binding protein J (CbpJ)	Choline binding, surface	36	88.97	Yes	Yes	Yes
SP_0390	spr0349		285	Choline binding protein G (CbpG)	Choline binding, surface	44	95.21	No	No	No
SP_0391	spr0350		340	Choline binding protein F (CbpF)	Choline binding, surface	16	90.33	Yes	Yes	Yes
SP_0462	Absent		893	RrgA pilus subunit, adhesin	LPXTG cell-wall anchored	8	91.66	No	No	No
SP_0463	Absent		665	RrgB pilus subunit, backbone	LPXTG cell-wall anchored	8	66.5	No	No	No
SP_0464	Absent		393	RrgC pilus subunit	LPXTG cell-wall anchored	8	99.09	No	No	No
SP_0498	spr0440		1,659	Endo-beta-N-acetylglucosaminidase, putative	LPXTG cell-wall anchored	44	97.02	Yes	Yes	Yes
SP_0641	spr0561		2,140	Serine protease, subtilase family	LPXTG cell-wall anchored	44	96.54	Yes	Yes	No
SP_0648	spr0565		2,233	Beta-galactosidase (BgaA)	LPXTG cell-wall anchored	44	96.54	Yes	Yes	No
SP_0664	spr0581		1,881	Zinc metalloprotease, putative (ZmpB)	LPXTG cell-wall anchored	43	71.13	Yes	No	Yes
SP_0667	spr0583		332	Pneumococcal surface protein, putative	Choline binding, surface	44	90	Yes	No	Yes
SP_0930	spr0831		627	Choline binding protein E (CbpE)	Choline binding, surface	44	96.64	Yes	No	No
SP_0965	spr0867		658	Endo-beta-N-acetylglucosaminidase (LytB)	Choline binding, surface	44	98.42	Yes	No	No
SP_0981	spr0884		313	Putative protease maturation protein (PpmA)	Lipoprotein	44	96.77	Yes	Yes	Yes
SP_1003	spr0907		839	Histidine triad protein	Lipoprotein	39	91.34	Yes	Yes	Yes
SP_1004	spr0908		1,039	Histidine triad protein	Lipoprotein	44	99.67	Yes	Yes	Yes
SP_1154	spr1042		2,004	Immunoglobulin A1 protease	LPXTG cell-wall anchored	44	79.85	Yes	Yes	No
SP_1174	Absent		819	Histidine triad protein	Lipoprotein	16	95.39	Yes	Yes	Yes
SP_1175	spr1061		802	Histidine triad protein	Lipoprotein	33	92.95	Yes	Yes	Yes
SP_1326	Absent		740	Putative neuraminidase (NanC)	Outer membrane/secreted	16	94.21	No	No	No
SP_1492	spr1345		202	Cell wall surface anchor family protein	LPXTG cell-wall anchored	44	91	Yes	Yes	Yes
SP_1572	spr1430		178	Non-heme iron-containing ferritin (PppA)	Surface	44	94.5	Yes	Yes	Yes
SP_1573	spr1431		490	Lysozyme (LytC)	Choline binding, surface	44	96.46	Yes	No	No
SP_1650	spr1494		309	Manganese ABC transporter, manganese-binding adhesion liprotein (PsaA)	Lipoprotein	44	97.92	Yes	Yes	Yes
SP_1687	spr1531		697	Neuraminidase B (NanB)	Surface	43	93.85	Yes	No	No
SP_1732	spr1577			Serine threonine kinase protein (StkP)	Membrane	44	95.33	Yes	Yes	Yes
SP_1772	Absent		4,776	Cell wall surface anchor family protein (PsrP)	LPXTG cell-wall anchored	14	99.7	No	No	No
SP_1833	spr1652		708	Cell wall surface anchor family protein	LPXTG cell-wall anchored	44	97.63	Yes	No	Yes
SP_1923	spr1739		471	Pneumolysin (Ply)	Secreted	44	99.5	Yes	No	No
SP_1937	spr1754		318	Autolysin (LytA)	Choline binding, surface	44	98.74	Yes	No	No
SP_1992	spr1806		221	Cell wall surface anchor family protein	LPXTG cell-wall anchored	44	99.01	No	No	No
SP_2136	spr1945		621	Choline binding protein PcpA (PcpA)	Choline binding, surface	32	99.05	Yes	No	No
SP_2190^c^	spr1995		693	Choline binding protein A (CbpA) (PspC)	Choline binding, surface	44		Yes	No	No
SP_2201	Absent		448	Choline binding protein D (CbpD)	Choline binding, surface	43	98.94	Yes	No	No
SP_2216	spr2021		392	Secreted 45 kDa protein (PcsB)	Secreted	44	96.75	Yes	Yes	Yes
Absent	Absent	SPNINV104_08710	410	PitB pilus 2 subunit, backbone	LPXTG cell-wall anchored	8	100	No	Yes	No
Absent	Absent	SPNINV104_08690	589	PitA pilus 2 subunit, ancillary	LPXTG cell-wall anchored	8	100	No	Yes	No

Due to the presence of unfinished genomes, we considered genes present in 43 *S. pneumoniae *genomes to be core. ^a^'Yes' indicates genes that are present in at least one of the strains of *S. mitis*, while 'no' indicates genes that are absent in the four analyzed strains. ^b^NanA is annotated as frameshifted in the TIGR4 strain, but it is known to be expressed by all pneumococcal strains. ^c^PspC has been classified in 11 major variants that cannot be aligned due to different gene organization.

Out of 47 selected proteins, 31 are part of the core genome of *S. pneumoniae *(since our dataset includes several draft genomes, we consider proteins present in 43 of 44 genomes to be conserved in all strains). Of these, 27 have an average percentage of identity greater than 90%, while the remaining are the well-known hypervariable proteins PspA, PspC, ZmpB and the IgA1 protease, four of the most important virulence factors expressed by *S. pneumoniae *[42-46].

**Figure 11:**
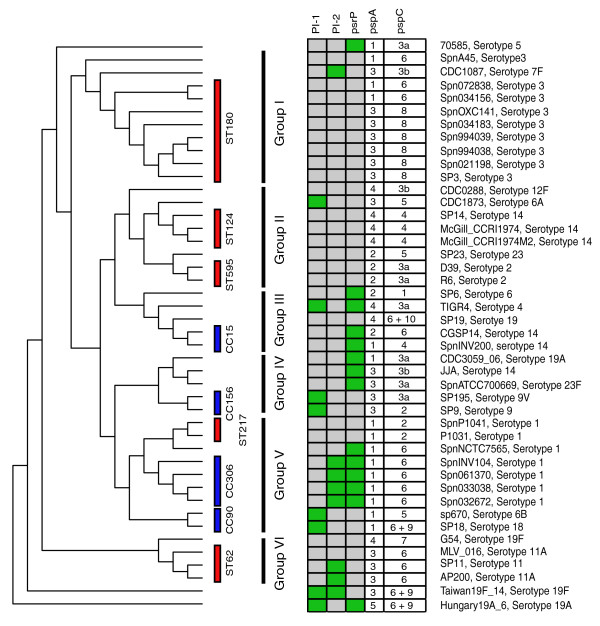
**Presence/absence pattern of the PI-1, PI-2 pilus-encoding islets, *psrP*, and allelic variants of the core *pspA *and *pspC *genes**. To show the degree of correlation with the phylogeny of *S. pneumoniae*, the data are reported on the phylogenetic tree of the *S. pneumoniae *strains. Only the topology of the tree is shown, branch lengths are not to scale. Red bars mark strains of the same ST, and blue bars mark strains of different STs, but of the same CC. For PI-1, PI2 and *psrP*, green squares indicate presence while gray squares indicate absence. For *pspA *and *pspC*, the numbers indicate the allelic variants defined according to [42,47].

According to sequence conservation, PspA is subdivided into three families, which in turn are classified into different clades: family 1 is composed of two clades (clade 1 and 2), family 2 comprises three clades (clades 3, 4 and 5), and family 3 has only one divergent clade (clade 6) [42]. Similarly, PspC has been classified in 11 major variants, based on sequence similarity and gene organization [47]. To verify whether the allelic profile of antigens correlates with pneumococcal phylogeny, we have mapped the allelic variants of PspA and PspC onto the tree of the species (Figure 11). As is generally found for most core genes, an overall strong association between allelic variants and MLST was observed, in agreement with recent data on PspA [48]. However, we found instances where a single ST or CC corresponds to more than one allelic variant (for example, the two antigens exist in two different variants within ST180 strains; similarly PspA,C in CC15, PspC in CC156, PspC in CC90), and little correlation was found with the lineages I to VI.

The remaining 16 proteins, including the structural components of the pneumococcal pili PI-1 (RrgA, RrgB and RrgC) and PI-2 (PitA and PitB), the serine-rich repeat protein PsrP [49], the putative neuraminidase NanC, five members of the choline binding proteins family (CbpC, I, J, F, and PcpA), the histidine triad proteins PhtA, D, E and the zinc metalloprotease ZmpC, were present in a variable number of strains (from 6 to 39).

The poor correlation between phylogeny and protein presence (Figure 11; Additional file 4) suggested that genes encoding proteins with antigenic properties might be acquired and lost easily. A good level of association was noted for PI-1 and PI-2 components, as previously reported [11,12], and for PsrP. According to a parsimonious reconstruction, the PI-1 islet appears to be present in the root of the *S. pneumoniae *tree, and to have been repeatedly lost during the course of evolution.

The patchy distribution of most of the surface-exposed proteins with antigenic properties is probably due to the selection exerted by the immune system, which selects for strains able to vary their repertoire of virulence-related genes. In the case of PI-1, evidence of selection driven by host immune response has recently been shown [50].

Furthermore, 34, 24 and 20 of the 47 putative virulence factors were also present in *S. mitis, S. oralis *and *S. infantis*, respectively, further supporting the concept that these commensals act as main gene reservoirs for *S. pneumoniae*. A closer look at the differences between *S. pneumoniae *and *S. mitis *revealed that some of the putative virulence factors that are always present and extremely conserved in *S. pneumoniae *are absent from all *S. mitis *strains. This group includes the hyalorunidase HysA, in agreement with the finding that *S. mitis *does not have hyaluronidase activity [1], StrH, which is involved in early colonization of the nasopharynx [51] and resistance to phagocytic killing [52], the choline binding protein G CbpG, and the two cell wall anchor proteins SP_0368 and SP_1992. Although the sampling of *S. mitis *is far from being exhaustive of the diversity of this species, this observation could suggest that these protein factors are crucial for pathogenicity.

## Conclusions

We have studied the genomic variability of *S. pneumoniae *in a large panel of isolates, including multiple serotypes and clonal complexes. Despite the effect of widespread recombination, the phylogeny inferred by a whole genome alignment is well supported and confirms the MLST-based classification. In particular, we found that strains of the same CC are highly correlated, both in terms of the composition of their dispensable genome and in the allelic variants of core genes. Given that the CCs represented in our collection account for 44% of the STs in the MLST database (56% excluding singletons), this study provides a good sample of the overall species diversity of *S. pneumoniae*. We identified six lineages broader than MLST classification that reveal more ancient relationships. However, since recombination events accumulate with time, these ancient groups are more affected than MLST-based CCs. As a consequence, the strength of the association with the composition of the genome is stronger for MLST than for lineage-based classification.

The dispensable genes are usually shared by small groups of strains and are frequently acquired and lost, suggesting that most of them are not fixed in the long term. This is compatible with previous analyses on the dispensable genome of *Escherichia coli*, where it was estimated that, on average, dispensable sequences are lost after 6.7 million years, to be compared with the 100 million years since the speciation of *E. coli *from *Salmonella enterica *[53].

The pan-genome of the *S. pneumoniae *species is open, and newly sequenced strains contribute a decreasing number of genes, as expected if the natural population of *S. pneumoniae *derived from a common ancestor. The longer an individual has evolved independently, the more new genes it will potentially contribute to the species pan-genome. The size of the pan-genome therefore reflects the degree to which the sequenced strains are phylogenetically related, and it yields information on the evolution of the species. A fast growing pan-genome, with strains that are quickly diversifying by integrating new genes, indicates that the species is exploring novel evolutionary possibilities. This process, we suppose, is only transient in the evolution of *S. pneumoniae *or any other species, since there are constraints on this diversification if the natural population is to remain a species. The position of *S. pneumoniae *on the extreme limit of being open is likely not due to chance. The species is possibly adapted to its current ecological niche, but is just open to the acquisition of new genes while maintaining stability. Analyzing the origin of dispensable sequences, we found that *S. mitis *is the main external reservoir of genetic variability of *S. pneumoniae*, and that most events of HGT involve closely related species that share a similar ecological niche.

Given these results, it is legitimate to ask why these organisms maintain a costly system to acquire, modify and lose genetic material. Experimental and simulation studies have shown that, under certain environmental conditions, intra-species homologous recombination increases the fitness of bacterial populations [54-56], at the same time limiting the divergence of lineages due to drift [57]. Similarly, it is conceivable that an open pan-genome, together with a mechanism to spread genes through unrelated strains, guarantees the species a quick and economical response to fluctuating environments.

These findings have obvious implications for vaccine design. Subunit vaccines based on variable genes with alleles that do not cross-protect might be subject to allele replacement. Similarly, dispensable genes used for vaccination might be discarded under the effect of immune pressure, unless they confer a selective advantage to the harboring strain. Although the estimated turnover timescale of the dispensable genome appears to be long, the selective pressure exerted by widespread vaccination could speed up these processes, as indicated by capsular switching events reported following the introduction of the heptavalent conjugate vaccine [58].

## Materials and methods

### Genome sequences

All genome sequences used in this study are listed in Additional file 1. They include complete genomes where all gaps remaining after assembly of shotgun reads have been closed. The TIGR4 genome sequence, the first one published [15], meets the absolute definition of a sequencing gold standard with all nucleotides covered by at least two reads, in most cases in opposite directions, all the repeated sequences confirmed for proper assembly, and all consensus bases inspected manually. Because this is an extremely time consuming effort, bringing all genomes to gold standard is not practically feasible. Thus, the other complete genomes do not meet the absolute gold standard but were nevertheless inspected for proper assembly and manually inspected for ambiguous consensus base calls.

The quality of draft genomes is highly dependent on the sequencing technology or technologies used and the amount of sequence coverage generated (Additional file 1). All the draft genomes used in this study display a minimum average sequence coverage per base of 10 ×. This combined with the requirement to identify SNPs shared by at least two genomes (see below) warrants that the SNPs used in this study are of high quality.

### Multiple genome alignments and identification of SNPs

Multiple genome alignments have been computed using the progressiveMauve algorithm of the Mauve software [59] using default options. Mauve produces an output file containing separate alignments for each LCB. We have extracted and concatenated each LCB containing sequences from each of the 44 genomes to build a core genome multiple alignment. From the concatenated multiple alignment the polymorphic sites were determined.

Polymorphic sites were identified from this core genome alignment as the loci where at least one sequence had a mutated base. We identified 79,171 polymorphic sites, of which 50,924 were informative, that is, the mutation was common to at least two sequences. While it is possible that some of the SNPs that are specific to a single sequence are due to sequencing errors, this possibility becomes very unlikely in the case of informative sites. Strain-specific SNPs can have an effect on the estimated value of the mutation rate and on the branch length of the phylogenetic tree. However, they do not affect the structure of the tree and the estimates of the recombination rate.

### Clonal complex designation

CCs were defined running eBurst [21] on the whole *S. pneumoniae *MLST database downloaded from the MLST website [60] in March 2009. Briefly, internal fragments of the *aroE, gdh, gki, recP, spi, xpt*, and *ddl *genes are sequenced and compared to alleles from the *S. pneumoniae *MLST website [60] for ST determination. In MLST, an ST is uniquely determined by the allelic profile. CCs are groups of STs that share a recent common ancestor. The eBURST algorithm [21] defines clonal complexes by partitioning the MLST data set into groups of single-locus variants, that is, profiles that differ at one of the seven MLST loci. This partitioning associates each ST with a CC and identifies the most likely founder ST, which is defined as being the ST with the greatest number of single-locus variants within the CC. Computed CCs were named after the ST of the predicted founder.

### Phylogenetic trees

Maximum likelihood phylogenetic trees were computed using RaxML [61] using the GTR model and discrete Gamma distributed rate parameters with four categories with bootstrap. RAxML first conducts a bootstrap search then identifies the best scoring tree with maximum likelihood and reports the support values for each branch of the best scoring tree. Branch support was computed by bootstrap with 1,000 replicates.

### Phylogenetic networks

Phylogenetic networks were generated using the program SplitsTree v.4 [24] using the NeighborNet method and distances computed with the Kimura 2 parameters substitution model.

### Clustering of orthologous genes

A complete description of the algorithms used to create the orthologous clusters is given by Hogg *et al*. [26]. Briefly, to allocate the genes into core or dispensable gene clusters, tfasty34 (Fasta package, version 3.4) was used for six-frame translation homology searches of all predicted proteins against all possible translations [62]. Software designed at the Center for Genomic Sciences was used to parse this output, grouping the genes into clusters that share 70% identity over 70% of the length with one or more of the other genes in the cluster. These parameters were selected because they minimize the change in the number of clusters per change in parameters and thus may represent a good estimate to distinguish orthologs from paralogs [26]. To account for the cases where genes were missed in the gene-calling step, fasta34 (Fasta package, version 3.4) was used to align all predicted genes against the contigs. If an alignment with the required score was detected in the contig, the gene was considered present even if the prediction software did not identify the gene.

### Allelic designation of core genes and association tests

The association between presence/absence of dispensable genes and the allelic form of core genes with the lineages I to VI and the CCs has been tested using the function 'assocstats' of the vcd v.1.2-3 package of the R statistical software [63]. Briefly, for the presence/absence profiles of each gene, we have computed the Cramer's V association index and the *P*-value of the Pearson correlation [22] with both the lineages defined in Figure 1 and the CCs. A value of V equal to 1 indicates perfect association between the partitions of the strains and the presence/absence, while V = 0 means that dispensable genes are randomly distributed amongst the groups. The *P*-values give the statistical significance of the association.

The allelic forms of the 680 selected core genes were assigned from the multiple alignments by giving a different ID to each different sequence. Similarly to the analysis performed for the presence/absence profiles of dispensable genes, for each gene we computed the Cramer's V and the *P*-value of the Pearson correlation between the allelic designation and the lineages and CCs.

### Identification of putatively recombining loci

For a given monophyletic group, we selected those of the 680 core genes that have at least two distinct alleles within the group, both of which are also present in other strains outside the group. In an infinite sites model it is guaranteed that, if the group is monophyletic, at least one of the two alleles has undergone recombination. This method provides a conservative estimate of the fraction of genes that underwent homologous recombination, since it cannot identify recombination where only a fraction of a gene is transferred and it is limited to cases in which both the parent and recombinant alleles are present in the dataset.

### Calculation of pan-genome size

#### Finite supragenome model

The finite supragenome model predicts the number of clusters of orthologous genes present in six population frequencies, varying from rare genes (less then 3% of the strains) to core genes (all the strains) [26]. The finite supragenome model trained on 44 sequenced strains identified a total of 3,221 clusters (Table 1) that were used to predict the number of core, dispensable and total clusters of orthologous genes expected for a 100-strain comparison. At 100 sequenced strains, the supragenome model predicts that 48% of the clusters are core and approximately 27% of the clusters are rare, that is, present in less than 3% of the strains.

#### Power law model

The average size Ω(*n*) of the pan-genome as a function of the number of strains *n *can be computed from the number of new genes *N*(*n*) found by sequencing the *n*-th genome by the following relation:

$$\Omega \left(n\right)=\Omega_{0}+\sum_{i=2}^{n}N\left(i\right)$$

where Ω_0 _is the average number of genes in a strain. By assuming *N*(*n*) = A*n^*ξ *^we obtain:

$$\Omega \left(n\right)≅\{\begin{array}{ccc} C_{1}+A_{1}n^{1+\xi } & \xi >-1 & \\ C_{2}+A_{2}\ln\left(n\right) & \xi =-1 & n\to \infty \\ A_{3} & \xi <-1 & \end{array}$$

where *A*_1_, *A*_2_, *A*_3_, *C*_1 _and *C*_2 _are constants. The pan-genome is 'open', that is, diverges as a power law for *n *→ ∞ for *ξ *> -1, logarithmically for *ξ *= -1, while it converges to a constant, that is, it is closed for *ξ *< -1.

### Dependency of the pan-genome size and the number of polymorphic sites

If we assume that each strain has a constant probability *ω *per unit of time to acquire a gene, the average number of new genes contributed by a strain to the pan-genome is proportional to the time *t*(*n*) since this has evolved independently from the *n *already sequenced strains. *t*(*n*), on average, decreases with *n*, since it is increasingly difficult to find new strains that are not closely related to the already sequenced ones. The average size Ω(*n*) of the pan-genome in a sample of n strains is given by:

$$\Omega \left(n\right)≅\Omega_{0}+\omega T\left(n\right)$$

where *T*(*n*) is the average length of the phylogenetic tree of the strains and Ω_0 _is the average size of a *S. pneumoniae *genome. If the tree distribution is well described by a coalescent process both with and without recombination, *t*(*n*) and *T*(*n*) are proportional to 1/*n *and 1n(*n*), respectively, for *n *→ ∞ [64], in agreement with our data (Figure 4 and Figure 5 inset). Similarly, the number of segregating sites in a set of n sequences is given by:

S(n)≅θT(n)

where *θ *is the population mutation rate. Substituting the average tree length, we obtain:

$$\Omega \left(n\right)≅\Omega_{0}+\frac{\omega }{\theta }S\left(n\right)$$

Therefore, by a linear fit of the relation between *S*(*n*) and Ω(*n*) we can estimate the ratio between the rate of acquisition of new genes and the mutation rate.

### Linkage disequilibrium

For each pair of polymorphic sites, we computed D' using the formula:

$$D'=\{\begin{array}{cc} \frac{D}{\min\left(p_{1},q_{2},p_{2}q_{1}\right)} & D>0\\ -\frac{D}{\min\left(p_{1}q_{1},p_{2}q_{2}\right)} & D<0 \end{array}$$

*D *= *p*_11_*p*_22 _- *p*_12_*p*_21 _where *p*_*ij *_is the proportion of individuals carrying allele *i *at site 1 and allele *j *at site 2, and *p*_*i*_,*q*_*i *_are the proportion of individuals carrying allele *i *at site 1 and 2, respectively. D' was then averaged over all pairs of polymorphic sites within a given alignment block as a function of their distance along the chromosome.

### Definition of the dispensable sequences

From the multiple genome alignment obtained using Mauve we selected 1,958 variable regions, that is, LCBs that are absent in one or more genomes and are longer than 500 bp, in order to avoid small regions where alignments could be problematic. Due to the alignment algorithm of Mauve, these regions are not split by indels in one or more of the genomes [59]. Of these regions, 1,030 are present in two or more strains and therefore are informative from the point of view of phylogenetic reconstruction.

### Genetic distances

The average genetic distance within and between the *S. pneumoniae *and the *S. mitis *groups was calculated by selecting the portions of the multiple genome alignment that were common to 50 genomes, concatenating those aligned sequences into a single multiple alignment and then computing on the concatenated sequences the pair-wise per-site number of substitutions using the maximum composite likelihood method [65] for the Tamura-Nei substitution model [66] using Mega 4.1 [67].

### Reconstruction of acquisition/loss events with parsimony

We represented the presence/absence of each of the 1,030 informative variable segments as a 0/1 character, and we computed the parsimony score *S*_*p *_of the tree for each of these characters. The parsimony score of a discrete character on a tree is the minimum number of state transitions that a character must have undergone if it evolved on a given tree, and in this case represents the minimum number of independent acquisition/loss events that are necessary for a given pattern of presence/absence. If the distribution of a given character can be explained by a single acquisition/loss, its parsimony score is 1, while if multiple events of acquisition/loss are necessary, its parsimony score is greater than 1 according to the rule *S*_*p *_= *N*_*a *_+ *N*_*l *_where *N*_*a *_and *N*_*l *_are the number of acquisitions and losses, respectively. Since an acquisition followed by a recombination event can always be explained by multiple acquisitions, events with *N*_*a *_> 1 are possible intra-species recombination events.

### Reconstruction of ancestral genomes

Ancestral genomes were reconstructed using the 'ace' function of the package ape v. 2.3 of the R software [68]. The presence/absence of each gene in a given strain was indicated as a two state discrete character (1 for presence, 0 for absence), and an asymmetric transition matrix between the two states with different acquisition/loss rates was assumed. The rates of acquisition/loss and the states in the internal nodes were determined by maximizing the likelihood of the observed distribution assuming that strains evolve on the three shown in Figure 1. A gene was considered to be present in a given node if the likelihood of the state '1' exceeded 0.5.

## Abbreviations

BP: base pair; CC: clonal complex; HGT: horizontal gene transfer; LCB: locally collinear block; MLST: multi-locus sequence type; SNP: single-nucleotide polymorphism; ST: sequence type.

## Authors' contributions

CD, NLH, AM and VM conceived the project, designed research, performed research, analyzed data, and wrote the manuscript; HT, SDB, MK, and ERM conceived the project, designed research, and wrote the manuscript; NJC performed research, analyzed data, and wrote the manuscript; HT, DRR, JCDH, SVA, FZH, NLH, and TJM contributed to shotgun sequencing, sequence assembly, annotation and database development; MO analyzed data and wrote the manuscript; DRR performed pan-genome analysis; GDE, AC, and RR wrote the manuscript.

## Supplementary Material

Additional file 1 **Table S1**. Click here for file

Additional file 2 **Supporting online materials**. Click here for file

Additional file 3 **Figure S1**. Click here for file

Additional file 4 **Table S2**. Click here for file

Additional file 5 **Figure S2**. Click here for file

Additional file 6 **Table S3**. Click here for file
