# Supplementary material for: Structure and dynamics of the pan-genome of Streptococcus pneumoniae and closely related species
Source: Genome Biol. 2010 Oct 29;11(10):R107. doi: 10.1186/gb-2010-11-10-r107 (PMC3218663; doi:10.1186/gb-2010-11-10-r107)
Supplement: Additional file 2 — Supporting online materials. [file gb-2010-11-10-r107-S2.DOC]

**Supporting Online Materials**

**Bayesian reconstruction of the whole-genome phylogenetic tree of *S. pneumoniae*.**

We have tested the reliability of the phylogenetic reconstruction shown in Fig. 1 using a Bayesian Markov Chain Monte Carlo method [69]. The obtained tree is shown in Fig. S1. The structure of the tree is similar to the structure of the tree in Fig. 1. The clades conserved between the two trees are indicated as lineage I-VI in Fig.1. The major differences are the positon of Group VI, which in this reconstruction appears to be closely related to clade I, and the position of the serotype 7F strain CDC1087, which does not belong to Group I, but instead belongs to a monophyletic brnch with the serotype 5 strain 70585. Using the possibility to define test statistics in the program BEAST [69], we have tested the probability that the six lineages I-VI are monophyletic. We obtain 100% probability for groups II-VI. For group I, the probability is 0 if we include also the strain CDC1087, and 100% if we only include the serotype 3 strains), suggesting that while the composition of lineages II-VI is robust, the inclusion of CDC1087 in lineage I depends on the tree reconstruction method.

**Whole genome phylogenetic analysis generalizes MLST-like typing methods**

To quantify the degree of correlation between phylogeny and dispensable genome, we measured the association between the lineages of Fig.1 and presence/absence of dispensable genes present in more than one strain (see also Table 1). For each of these genes, we quantified the strength of the association between the allelic variant and the lineages of Fig.1 by computing the association index V (see Methods). A value of V=1 means perfect association, while V=0 indicates random association between allelic variants and lineages. The average association value was 0.5 (median of the P-values equal to 0.07) indicating, in most cases, a non-random association between presence/absence of dispensable genes and lineages. Repeating the analysis for the association of the dispensable genome with the CCs we found an average value of V=0.82 (median of the P-values equal to 0.007).

Given the extent of homologous recombination, we investigated the extent to which the allelic variants of core genes correlated with lineages I-VI inferred by molecular phylogenetics. We identified 680 core genes that were present in single copy in each genome, and classified the distinct alleles of each of these genes. The average association is 0.747 (median of the P-values equal to ) indicating a strong association between alleles and lineages. Not surprisingly since CCs are always contained in a single lineage, their association was even stronger (V equal to 0.94, median of the P-values equal to $5.3\times10^{-11}$). We conclude that, knowing the CC of a strain, we can predict the allelic variant of most core genes with high level of confidence.

Taken together, these results indicate that there is a strong and statistically significant association between the allelic form of core genes and the classification into lineages I-VI and CCs. A significant association exists also between presence/absence of dispensable genes and both lineages and CCs. This association is weaker than in the case of allelic variants of core genes, probably due to the more dynamical nature of the dispensable genome, which is rich in mobile elements [3].

**Clustering based on presence/absence of dispensable genes.** The pattern of presence/absence can be used to obtain a hierarchical clustering, that identifies groups of related strains. In Fig.S8 we show a hierarchical clustering based on presence-absence of dispensable regions; red boxes mark the cluster exceeding the 98% confidence level using a multiscale bootstrap resampling method [70, 71]. The clusters obtained with this method coincide with monophyletic branches of the phylogenetic trees shown in Fig.1, confirming the correlation between genomic content and genetic distance. Compared to Fig. 1, there are variations in the mutual position of some groups. For instance, Group V containing all serotype 1 strains is split by this analysis into tree distinct clusters, one corresponding to the CC306 strains, the second formed by the CC90 strains 670_6B and SP18 whose dispensable genome is more closely related to the Taiwan19F_14 and G54 strains, and the third corresponding to the CC217/CC2296 strains, that are more strictly related to the serotype 3 strains. Interestingly this is confirmed by an analysis of the repeats present in these genomes, because NCTC7465, P1031 and P1041 share some repeat families that are only found in the serotype 3 isolates in this dataset (see below). Group I, containing all serotype 3 strains and the serotype 7F CDC1087 strain, has been split in a group containing only the serotype 3 strains, while the strain CDC1087 is not strictly related to any other strain, suggesting that, as indicated also by the Bayesian posterior probability, the relative order of the A45 and CDC1087 branches in Fig.1 is probably an artifact of the phylogeny reconstruction method.

**Insertion Sequences are more variable than other phylogenetic markers.**

In total, intact representatives of 19 different insertion sequence (IS) types could be identified in the dataset. This includes ISSpn7, found to have caused an insertion disrupting the hyaluronate lyase gene of *S. pneumoniae* strain SP18, and ISSpn8. The frequency distribution of IS elements is variable. For instance, we found that IS200 is present as a single copy (either intact or degenerate) in each genome, and there were no observed instances of transposition. In contrast, other elements, such as ISSpn5, were absent from some isolates, but increased the copy number in others. It seems likely that this dissemination within genomes occurs relatively quickly compared to other changes in the genome, e.g. alterations in the sequence of the MLST loci. For example, IS1515 in the serotype 1 genome Sp033038 and Sp032672 contain 16 and 14 copies of this transposon respectively, whilst strain Sp061370 (of the same ST) has only one copy, suggesting that loss or gain of IS1515 has occurred since these strains have diverged from each other. However, there is little phylogenetic signal in the distribution of repeats, which is likely to be a consequence of the high turnover of such elements: the majority of CDS annotated as being transposons appear to be non-functional, suggesting they are remnants of past IS expansions. Hence the IS complement of each pneumococcal chromosome appears to be in a state of flux, with new families imported and expanding to replace those that are eroded by inactivating mutations.

Fig.S1. Bayesian phylogenetic tree obtained using the SNPs of the whole genome alignment of the 44 genomes of *S.pneumoniae*.

Fig. S2 Hierarchical clustering of the stains based on the presence/absence of dispensable regions. Red numbers indicate the percentage p-value of the branch computed using the Approximate Umbiased (AU) method (see Methods), while green numbers indicate the bootstrap support. Blue and red bars indicate MLST Clonal Complexes and Sequence Types, respectively.

Table S1. Sequenced strains used in this study. For each strain, we report name, genome length, number of predicted coding sequences, locus of isolation, disease or carriage status, sequencing status, source of the sequence, accession number, sequencing technology and average sequence coverage. For pneumococcal strains, also ST and CC determined by the eBURST algorithm [21] are reported. *)Finished genomes are complete genomes that were inspected for proper assembly and manually inspected for ambiguous consensus base calls.**)These Whole Genome Shotgun projects have been deposited at DDBJ/EMBL/GenBank under the accession xxxx00000000. The version described in this paper is the first version, xxxx01000000

Table S2. For each strain (rows) we report the number of genes not conserved in the other sequenced genomes (columns).

Table S3. Distribution of the genes from Table 3 which are not shared by all *S. pneumoniae* strains. Green squares indicate presence, gray squares indicate absence of the gene.
