# Supplementary figures and images for: Structure and dynamics of the pan-genome of Streptococcus pneumoniae and closely related species
Source: Genome Biol. 2010 Oct 29;11(10):R107. doi: 10.1186/gb-2010-11-10-r107 (PMC3218663; doi:10.1186/gb-2010-11-10-r107)

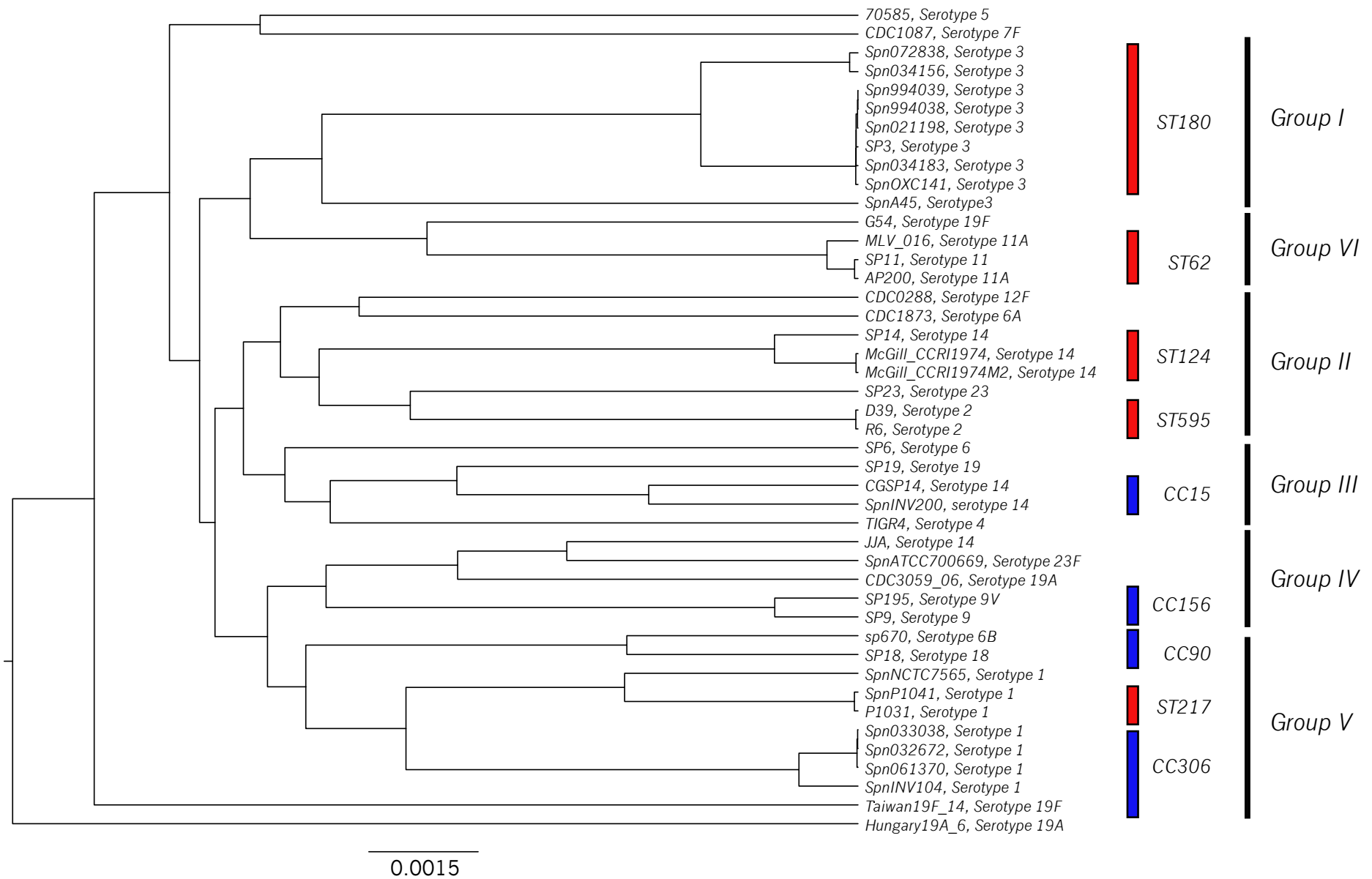

Supplement: Additional file 3 — Figure S1. [file gb-2010-11-10-r107-S3.PDF]

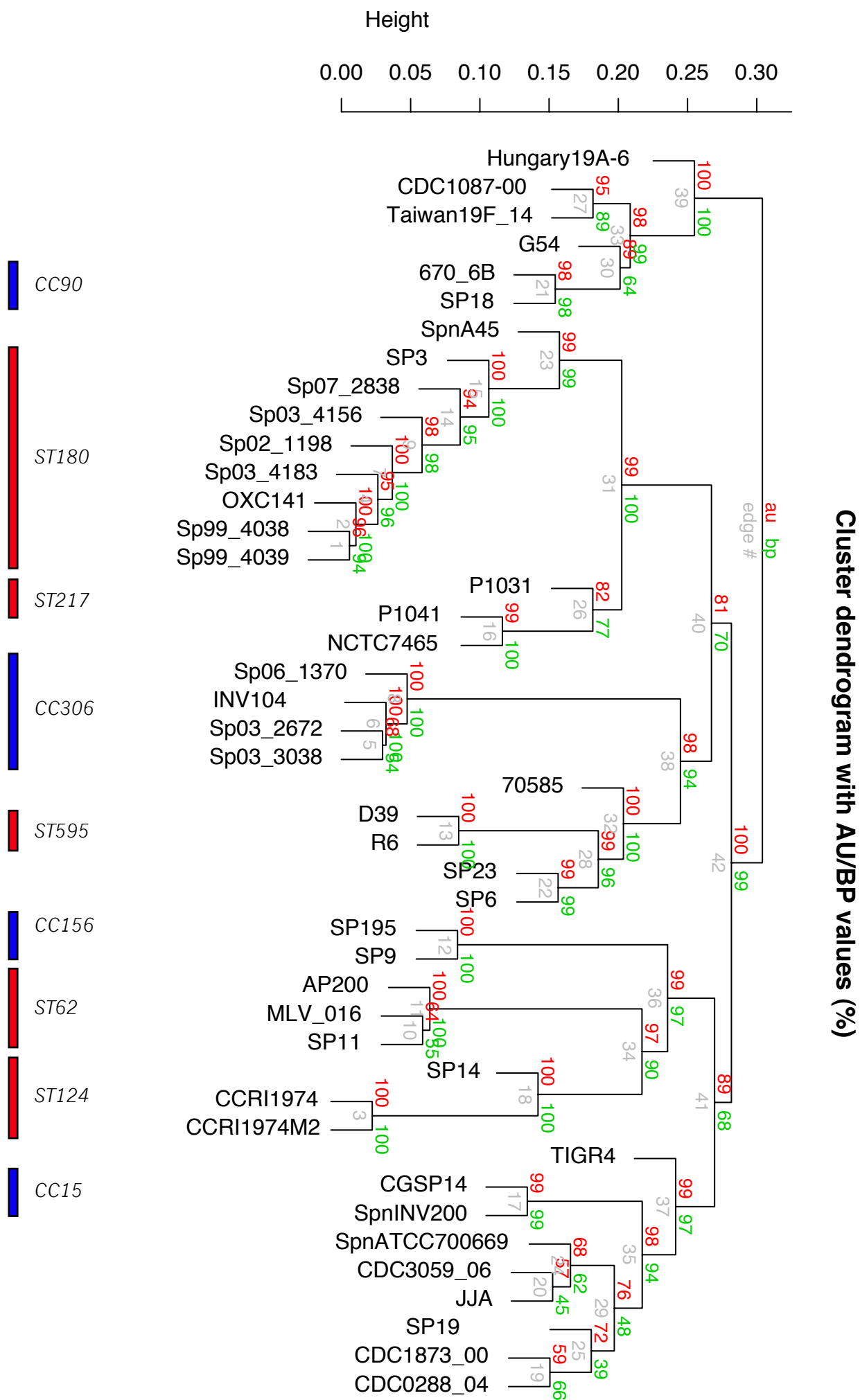

Supplement: Additional file 5 — Figure S2. [file gb-2010-11-10-r107-S5.PDF]
